# The value of diagnostic ultrasonography in the assessment of a glomus tumor of the subcutaneous layer of the forearm mimicking a hemangioma: a case report

**DOI:** 10.1186/s13256-015-0672-y

**Published:** 2015-09-09

**Authors:** Dong-Yeong Lee, Sun-Chul Hwang, Soon-Taek Jeong, Dae-Cheol Nam, Jin-Sung Park, Jeong-Hee Lee, Jae-Boem Na, Dong-Hee Kim

**Affiliations:** 1 Department of Orthopaedic Surgery, Research Institute of Clinical Medicine, Institute of Health Sciences, Gyeongsang National University School of Medicine, Jinju, Republic of Korea; 2 Department of Pathology, Gyeongsang National University School of Medicine, Jinju, Republic of Korea; 3 Department of Radiology, Gyeongsang National University School of Medicine, Jinju, Republic of Korea; 4 Department of Orthopaedic Surgery, Gyeongsang National University School of Medicine and Gyeongsang National University Hospital, 15, Jinju-daero 816 beon-gil, Jinju-si, Gyeongsangnam-do 660-751 Republic of Korea

## Abstract

**Introduction:**

A glomus tumor is a rare, benign tumor with atypical clinical symptoms. Because of its small size, it is difficult to diagnose and treat early; therefore, it leads to poor quality of life. Glomus tumors are known to commonly affect the hand and rarely manifest in other areas. Because they simulate neuromas, hemangiomas, and neurofibromatosis, the differential diagnosis is difficult. We performed marginal resection of a solitary forearm mass previously suspected to be a hemangioma or glomus tumor on the basis of ultrasound findings and histologically diagnosed to be a glomus tumor afterward. We report this case to demonstrate the good prognosis of the procedure we used, and we review the relevant literature.

**Case presentation:**

A 68-year-old Asian man without a particular medical history visited our hospital with a mass with focal tenderness in his left distal forearm that had developed 8 years earlier. The tumor was observed with suspicion of being a hemangioma or glomus tumor based on the location, clinical symptoms, and ultrasound findings taken into consideration together. The biopsy results led us to conclude that the lesion was a glomus tumor.

**Conclusions:**

A glomus tumor located in the forearm is very rare. It is often clinically overlooked and is likely to be misdiagnosed as another disease. The patient’s quality of life deteriorates, and, though the disease is rare, it has serious sequelae. Therefore, a quick diagnosis and appropriate treatment must be conducted early. If a mass occurs with serious pain in subcutaneous soft tissue of not the hands but the limbs, it is important to conduct examinations with suspicion of a glomus tumor. Ultrasonography performed quickly may be useful for making the differential diagnosis.

## Introduction

The glomus tumor was first reported to be a “painful subcutaneous nodule” in 1812 by Wood, and it was later named *glomus tumor* based on anatomic and pathologic findings by Masson in 1924 [1]. The glomus body is located in the reticular layer of the dermis in a special form of arteriovenous anastomosis without a connection to capillaries, and it is widely distributed over the body surface, but mostly in the hands [1]. A glomus tumor occupies 1.6% of an entire soft tissue tumor, and its presence in areas other than the hands is very rare. It mainly affects men in their 30s to 60s [2]. Typically, a glomus tumor manifests with three major symptoms (pain, tenderness, and cold hypersensitivity) that do not always appear together. The size of a glomus tumor is about 5mm, and most are less than 10mm, so it is very difficult to diagnose one that occurs in a site other than the hands [3]. In addition, patients are often misdiagnosed with, for example, hemangioma, neuroma, neurofibromatosis, or lymphadenitis and receive treatment for these, leading to a delay in the correct diagnosis [4]. The treatment is complete surgical resection, which is known to be effective. The disease is rarely reported to be recurrent, but this is known to happen following incomplete resection [2].

We performed a complete resection of a glomus tumor in a patient who had a tumor accompanied by focal tenderness in his forearm. His tumor was suspected of being a hemangioma or glomus tumor based on severe pain according to the location of the tumor, his clinical symptoms, and the results of ultrasonography. He was definitively diagnosed with a glomus tumor based on a pathologic examination. We report his case and review the relevant literature.

## Case presentation

**Fig. 1:**
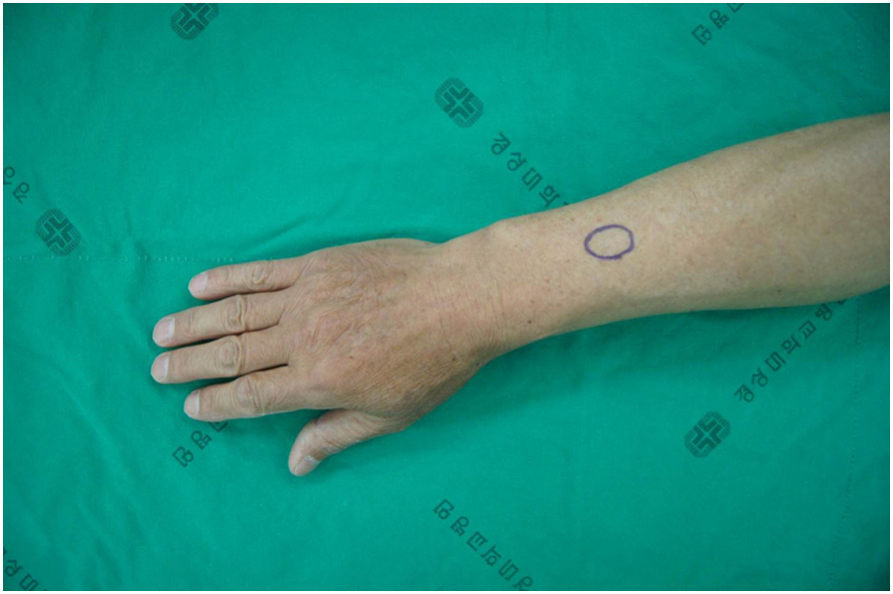
Pre-operative clinical photograph shows a vague, mass-like lesion that had a definite local tender point on the dorsal aspect of the forearm

**Fig. 2:**
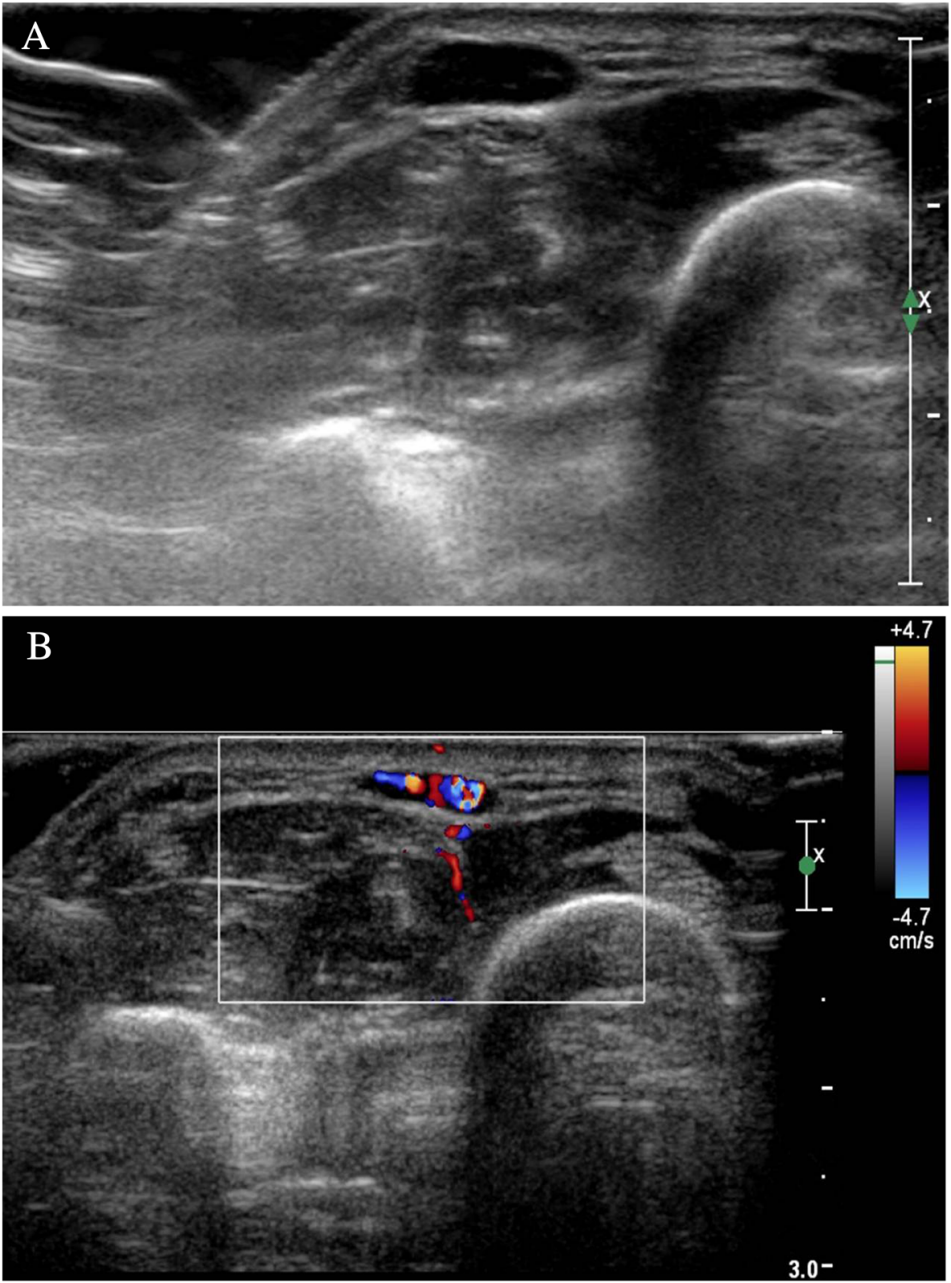
Ultrasonograms of the mass lesion. **a** Ultrasonogram of the forearm shows a well-defined, hypoechoic, 0.9cm soft tissue nodule. **b** Soft tissue mass with increased vascularity

A 68-year-old Asian man without a particular medical history visited our hospital with a complaint of a mass with focal tenderness that had developed 8 years earlier in his left distal forearm. He did not complain of cold hypersensitivity (worsening of pain when exposed to coldness), but he had positive findings for Tinel’s sign accompanied by serious numbness upon percussion. The mass had a round, soft surface of about 0.8cm in macroscopic appearance, and it was movable within a narrow range (Fig. 1). The patient did not have motor weakness, radiating pain, or a sensory change in the distal mass. He had no particular family medical history, and he had no pain or discomfort unless the tumor was pressed. Hematologic testing and plain radiography performed in the hospital produced no special findings. High-resolution ultrasonography was done to identify the size, accurate location, and patterns of the tumor. Magnetic resonance imaging (MRI) was not performed, owing to financial reasons expressed by the patient. A 0.9cm nodule was observed on an ultrasound. It was located in the subcutaneous fat layer of the forearm. It was a well-defined hypoechoic tumor with slight acoustic enhancement. The blood vessel was observed to be included inside the lesion. Hypervascularity was observed in the tumor on a Doppler ultrasound, which made us suspect a hemangioma or glomus tumor on the basis of the location, clinical symptoms, and ultrasound findings together (Fig. 2).

**Fig. 3:**
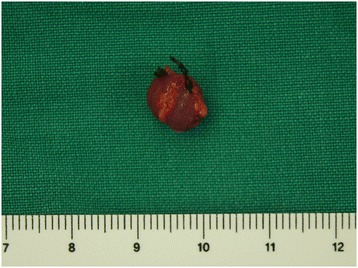
Intra-operative photograph shows a dark red solid tumor. The tumor is well-defined and sheathed within a fibrous capsule, and it has a feeder vessel (black ties)

**Fig. 4:**
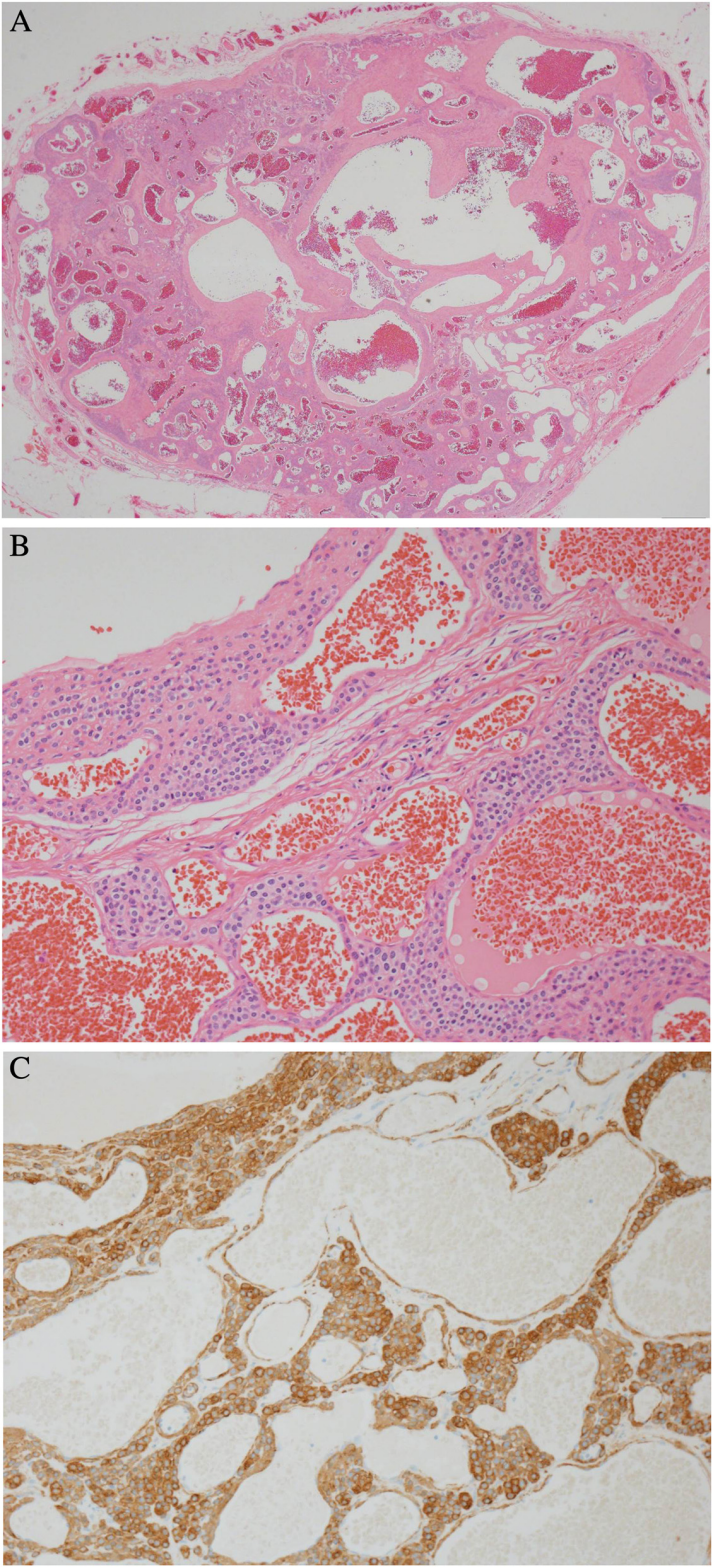
Microscopic findings of the mass lesion. **a** The tumor shows a circumscribed tumor composed of scattered and dilated blood space (hematoxylin and eosin stain; original magnification, ×20). **b** Stained specimen shows blood vessels lined by normal endothelial cells and round to ovoid cells with round nuclei and acidophilic cytoplasm (hematoxylin and eosin stain; original magnification, ×200). **c** Tumor cells are smooth muscle actin–positive (original magnification, ×200)

Surgery was performed with the patient under local anesthesia. A 1cm longitudinal incision was made in the location of the tumor to identify the dark red solid tumor surrounded by a fibrous capsule distinguishable from surrounding tissues. During the resection, a small vessel that supplied the inside of the tumor was identified and ligated. After the tumor was removed, the patient was discharged from the hospital on the same day (Fig. 3). The biopsy findings were that the size of the excised tumor was 0.8cm×0.6cm×0.4cm, the vascular tissues expanded to the tumor surrounded by the fibrous capsule were scattered, and tumor cells with eosinophilic cytoplasm containing equal nuclei were visualized by hematoxylin and eosin staining under an optical microscope (Fig. 4). Round, epithelioid cells proliferated around capillaries consisting of normal endothelial cells and sheets. Therefore, immunochemistry was additionally conducted because of pathologic findings suggestive of a glomus tumor rather than a hemangioma, which had been expected prior to the operation. The tumor cells were found to be smooth muscle actin–positive and CD34-positive, which definitively indicated a glomus tumor. The patient’s severe pain due to tenderness of tumor disappeared completely, and the he recovered well without other complications or signs of recurrence.

## Discussion

According to the Mayo Clinic, a glomus tumor is a rare benign tumor that accounts for 1.6% of soft tissue tumors occurring in limbs and about 2% of hand tumors. It is reported that 25–65% of glomus tumors occur in the subungual region [5, 6]. In addition to developing in the hands, glomus tumors may occur in all other areas of the body with a normal glomus body, and their occurrence has been reported even in areas without a normal glomus body. The conversion to a malignant tumor is reported to be very rare, less than about 1% [7].

Typical symptoms of glomus tumors include pain, tenderness, and cold hypersensitivity. However, because all of these three symptoms are not observed simultaneously in all glomus tumors, the symptoms are not that helpful for making a diagnosis [5]. In addition to the above-described symptoms, a number of other ones, including tingling pain, causalgia, and radiating pain, may occur; therefore, it is important to suspect glomus tumors in patients who complain of these symptoms. In our patient, the main symptom he reported was only tenderness among the three symptoms of glomus tumors, and the tumor was located not in the hands, which the glomus tumor often affects, but in the forearm. Because the tumor size was very small, and because it presented without skin discoloration, it was difficult to suspect a glomus tumor until the biopsy result was identified. Also, the patient had not visited the hospital when he first noticed his symptoms, because he had no particular pain unless the lesion was pressed, and he had been careful not to press the lesion to prevent pain in his everyday life. He was diagnosed 8 years after the first symptom occurrence.

It is important for early diagnosis and treatment to suspect a glomus tumor in patients who complain of the above-described symptoms in areas other than the hands. Generally, a glomus tumor is reported to be treated well with complete resection, and the rate of recurrence is reported to be under 10%. Recurrence is caused by incomplete resection [2]. In addition, the possibilities of malignant change and distant metastasis have also been reported, implying that continuous follow-up is needed. Because deterioration of a patient’s quality of life and the risk of complications are expected owing to such properties of a glomus tumor, it is important to suspect the disease early and initiate proper treatment.

Most of the conventional radiologic findings on our patient’s imaging examinations were normal, but cortical bone breakage or cystic bone breakage caused by the pressure of the tumor may be seen in some patients. If the clinical symptom of cold hypersensitivity does not exist when the lesion occurs in areas other than those the lesion generally affects, it will be even more difficult to suspect a glomus tumor. In that situation, a glomus tumor must be differentiated from other vascular tumors, such as hemangiomas or vascular malformations, as we did in our patient. Indeed, the term *hemangioma* was originally used to describe any vascular tumor-like structure, whether it was present at or around birth or appeared later in life. The classifications and terminology used for various vascular lesions can be very confusing, despite the fact that a biological classification was first published in 1982 by Mulliken and Glowacki [8]. On ultrasounds, vascular tumors such as hemangiomas or vascular malformations, hypoechoic or hyperechoic patterns may occur, posterior acoustic shadowing can be observed, and ill-defined margins with adjacent tissues may occur frequently. The blood flow rate in vascular tumors occurs in various ways, but a hypervascular tumor pattern may been seen in some cases. It may be observed that the blood flow of vascular tumors in a subcutaneous fat layer decreases owing to the pressure on the vein inside the tumor but increases again when pressure is released. Detection of this symptom helps in making the correct diagnosis [9]. According to Mulliken and Glowacki [8], however, hemangiomas are characterized clinically by appearing at or shortly after birth. A rapid proliferative phase occurs during the first 9–12 months of life, followed by an involution phase that may be completed by 3–5 years of age; but this phase can last up to 12 years of age. In addition, vascular malformations that are present since birth tend to grow proportionately with the patient’s age. Therefore, we considered vascular tumors such as a hemangioma or a vascular malformation to be unlikely in our patient. On the contrary, a glomus tumor shows hypoechoic findings and posterior acoustic enhancement on ultrasonograms. Despite its very small size, usually less than 1cm, its boundary with adjacent tissues is clear. With a glomus tumor around cortical bone, erosion or breakage of the cortical bone may be observed. Also, in Doppler sonography, a glomus tumor shows hypervascularity, which is accompanied by severe pain rather than a change of shape due to pressure [8]. Thus, in patients with such findings, it is of great help to make a diagnosis of a glomus tumor. Ultrasonography is useful in differentiating the tumor and identifying its location and size, but it needs careful attention because it may appear negative for lesions as small as 2mm. In such patients, MRI may be helpful. For glomus tumors, T1-weighted sequences show intermediate or low signal intensity, similar in degree to muscle; T2-weighted sequences show high signal intensity; and contrast-enhanced images shows high signal intensity [4]. In our patient, no specific findings were observed on conventional radiographs; the boundary with surrounding tissues was found to be clear on ultrasound; the proliferation of blood vessels was observed as a hypoechoic mass located inside the subcutaneous fat layer; and increased blood flow was visualized by Doppler sonography. MRI could not be performed in our patient, owing to his expressed financial reasons. Considering that the only clinical symptom was tenderness and the tumor was located in an area other than the hands, the typical occurrence site for glomus tumor, it was difficult to differentiate a hemangioma from a glomus tumor. However, on the basis of our ultrasound findings, a glomus tumor rather than a hemangioma was suspected, and a surgical resection was performed. The diagnosis of a glomus tumor was ultimately made on the basis of post-surgical pathologic findings. After complete resection, the patient fully recovered without additional treatment, and his pre-operative clinical symptoms disappeared completely immediately after surgery and during follow-up.

## Conclusions

A glomus tumor in the forearm is very rare. It is often clinically overlooked, is likely to be misdiagnosed as another disease, diminishes the patient’s quality of life, and, though rarely, has serious sequelae. Therefore, a quick diagnosis must be made and appropriate treatment must be initiated early. If a mass occurs with serious pain in subcutaneous soft tissue of not the hands but the limbs, it is important to conduct examinations with suspicion of disease glomus tumor. It is thought that ultrasonography, which can be performed quickly, may be useful for differential diagnosis.

## Consent

Written informed consent was obtained from the patient for publication of this case report and any accompanying images. A copy of the written consent is available for review by the Editor-in Chief of this journal.

## Abbreviations

MRI: Magnetic resonance imaging
